# Community pharmacists’ knowledge, attitude and practices towards the use of complementary and alternative medicines in Durban, South Africa

**DOI:** 10.4102/hsag.v24i0.1029

**Published:** 2019-03-18

**Authors:** Yasmeen Thandar, Julia Botha, Anisa Mosam

**Affiliations:** 1 Department of Basic Medical Sciences, Durban University of Technology, Durban, South Africa; 2 Discipline of Pharmaceutical Sciences, School of Pharmacy and Pharmacology, University of KwaZulu-Natal, Durban, South Africa; 3 Department of Dermatology, University of KwaZulu-Natal, Durban, South Africa; 4 Nelson R. Mandela School of Medicine, University of KwaZulu-Natal, Durban, South Africa

## Abstract

**Background:**

Atopic eczema (AE) is a common skin disease with an increasing worldwide prevalence, which has almost doubled over the last decade in South Africa. Many patients commonly explore complementary and alternative medicines (CAM) for AE and often initially seek advice from their local pharmacists.

**Aim:**

To explore the knowledge, attitude and practices amongst community pharmacists regarding CAM.

**Setting:**

The study was conducted amongst pharmacists working in community pharmacies in Durban, South Africa.

**Methods:**

During 2016, a cross-sectional study was conducted amongst 158 randomly selected pharmacists, of which 82 responded. Respondents were sent an email with a link to the questionnaire. Where logistically possible, questionnaires were hand-delivered.

**Results:**

The majority of respondents were male (*n* = 46; 56%), aged between 31 and 40 years. Despite most pharmacists not being familiar with various CAMs for AE, many (43%) recommend them, and 50% were amenable to referring patients to CAM practitioners. Despite 51% reporting that patients do ask about CAM for AE, 54% are not confident discussing or initiating discussions with patients. More than half of the pharmacists (55%) had no CAM training but believed it is essential for inclusion in the undergraduate pharmacy curriculum. Most were interested in broadening their knowledge on CAM and felt it would better prepare them in counselling their patients.

**Conclusions:**

The study demonstrated poor knowledge and communication about CAM for AE between pharmacists and patients, although pharmacists exhibited strong interests in learning more about CAM. There is a continuing need for education programmes and inclusion into undergraduate curricula that would assist pharmacists to advise patients on different types of CAMs.

## Introduction

Atopic eczema (AE) is a common skin disease with an increasing prevalence in many parts of the world, including Asia, Europe and Africa. Its prevalence has almost doubled over the last decade in South Africa (Deckers et al. 2012). The chronic nature of the disease and its clinical incurability, despite an array of effective mainstream treatment options available, drive AE patients to explore complementary and alternative medicine (CAM) with the hope of relief. Evidently, a recent South African study showed that 66% of patients had reportedly used CAM for AE (Thandar et al. 2017).

The Health Products Association of South Africa (an association of manufacturers, importers and distributors of complementary medicines and health products) estimated that 50% of turnover in complementary medicines occurred in pharmacies, 20% in health food stores and the balance in supermarkets, chain stores and toiletry discount outlets (Gqaleni et al. 2007). Consequently, pharmacies are the major stockists of CAM products, and it is where many patients seek guidance on their use.

A related survey in Australia conducted by Braun et al. (2010) showed that more than 90% of customers expect the pharmacist to recommend efficacious CAMs and to provide them with information with regard to their safe usage (Braun et al. 2010). Furthermore, Australian studies have demonstrated that while pharmacists accept responsibility for ensuring the safe use of CAM, many have the impression that they lack the knowledge and education to properly assist patients (Culverhouse & Wohlmuth 2012; Kanjanarach, Krass & Cumming 2011).

A South African study encompassing the attitudes and knowledge of pharmacists towards the use of herbal medicines in particular revealed that although know-ledgeable, 85% reported that they did not feel competent enough to adequately advise patients on the safe, effective and rational use of herbal medicines (Brijlal et al. 2011). While there are several international studies that focus on knowledge, attitudes and practices amongst pharmacists concerning the use of CAM (Alkharfy 2010; Bushett et al. 2011; Naidu, Wilkinson & Simpson 2005), none has focused on their use within the context of a specific disease nor has been conducted in a South African setting or within the African continent.

Considering the increased prevalence of AE in South Africa and the extensive use of CAM by South African patients for this particular disease, the perspectives of pharmacists regarding their use of CAMs is of great importance.

## Methods

### Study design

The researchers conducted a cross-sectional, questionnaire-based study in 2016 amongst community pharmacists practicing within the Durban area, South Africa, with the aim of exploring their knowledge, attitude and practices concerning CAM for AE.

### Study population

Practising pharmacists within the Durban area, South Africa, were recruited into the study using the random selection method from professional societies’ databases and the telephone directory. The sample size of 158 was calculated based on population estimates of practising retail pharmacists to properly represent the number of practising pharmacists in Durban and surrounding areas (within a 20 km radius of Durban).

### Inclusion and exclusion criteria

All pharmacists had to be practising in a private practice where AE patients would consult them for treatment. Practising pharmacists within retail pharmacies were included in the study. Hospital pharmacists with no retail components, as well as academic and industrial pharmacists, were excluded from the study. Respondents were recruited between October 2014 and February 2015. A total of 158 retail pharmacists were selected, of which 82 responded to the survey (52% response rate).

### Data collection

Respondents were sent a mail electronically with a link to the questionnaire. Where possible, questionnaires were hand-delivered to pharmacists practising nearby. Three reminders were sent electronically and through telephone calls. Written informed consent was obtained from all pharmacists.

The questionnaire was divided into the following sections:

- Practitioner and practice particulars
- General and demographic particulars of the health care practitioner (HCP)
- Views or attitudes on CAM for AE
- Knowledge of CAM for AE
- Professional practices regarding CAM
- Education regarding CAM for AE.

### Statistical analyses

Stata 13.0 (Stata Corp. 2013. Stata Statistical Software: Release 13. College Station, TX: Stata Corp LP) was used to analyse the data. Categorical data were summarised using frequencies and percentages. Association between the pharmacists’ attitudes, familiarity and practice using CAM variables were assessed using the Pearson chi-square (χ^2^) test and Fisher’s exact test (if any cell count contained fewer than five expected observations). A *p*-value < 0.05 was considered as being statistically significant.

### Ethical considerations

The researchers further confirm that any aspect of the work covered in this manuscript that has involved human respondents has been conducted with the ethical approval provided by the Biomedical Research Ethics Committee at the University of KwaZulu-Natal (BE 219/14) for the duration of this study and that such approvals are acknowledged within the manuscript.

## Results

### Demographics of pharmacists

A total of 82 pharmacists completed the survey questionnaires. The study population demographics are shown in Table 1.

**TABLE 1:** Demographics of Durban community pharmacists who participated in the survey (*n* = 82).

Demographic	*n*	%
**Gender** Males Females	46 36	56 44
**Age (years)** < 30 31–40 41–50 > 50	9 31 23 19	11 38 28 23
**Race** Black South African White South African Indian	6 15 61	7 18 74
**Religion** Christian Hindu Muslim Atheist/Agnostic/Undisclosed	22 27 29 4	27 33 35 5

### Knowledge and attitudes

The researchers assessed the views and attitudes of pharmacists towards CAM based on their degree of agreement or disagreement with various statements surrounding their use. Most pharmacists were generally neutral towards the use of CAM by patients. Almost half (46%) disagreed that the effect of CAM is because of the placebo effect, and the majority (59%) agreed that better clinical outcomes are produced by practitioners who are knowledgeable in CAM practices. Approximately half disagreed with the assertion that fraudulent claims are made about CAMs and that they interfere with standard medical care (51% and 49%, respectively). Table 2 shows the pharmacists’ attitude towards CAM.

**TABLE 2:** Durban community pharmacists’ views/attitudes towards complementary and alternative medicines for atopic eczema.

Structured questions	Agree (%)	Neutral (%)	Disagree (%)
1. CAM provides a more holistic approach to health than conventional medicines.	29	49	22
2. Most CAMs are safe and have very few side effects.	37	40	23
3. CAM can offer patients benefits that conventional medicines cannot.	35	41	23
4. The results of complementary therapies are because of the placebo effect.	11	43	46
5. Patients whose physicians are knowledgeable about CAM practices, in addition to conventional medicine, have better clinical outcomes.	59	34	7
6. Physicians should have knowledge about the most prominent CAM treatments.	83	12	5
7. CAM therapies should be subjected to more scientific testing before being accepted by conventional doctors.	87	12	1
8. CAM can produce longer lasting and more complete clinical results than conventional medicines.	11	55	34
9. I am annoyed when I find out my patients are using CAM without telling me.	16	34	50
10. CAM means quackery and makes fraudulent claims.	10	39	51
11. Interferes with standard medical care.	15	37	49

CAM, complementary and alternative medicines.

### Familiarity with complementary and alternative medicines

The pharmacists showed very little familiarity regarding the specific CAM practices. Probiotics and dietary supplements were the most well-known (45% and 33%, respectively). Chinese herbal medicine (CHM) was by far the least known (84% unfamiliar). The familiarities to all CAM are shown in Table 3.

**TABLE 3:** Durban community pharmacists’ familiarity with complementary and alternative medicines for atopic eczema.

Types of CAM	Unfamiliar (%)	Slightly familiar (%)	Very familiar (%)
1. Homeopathy	41	48	11
2. Chinese herbal medicine	84	12	4
3. Probiotics	22	33	45
4. Dietary supplements	13	54	33
5. Oral herbal products	17	57	26
6. Topical herbal creams	17	61	22

CAM, complementary and alternative medicines.

### Practices

The current practices of pharmacists regarding CAM show that more than half (54%) of the respondents rarely or never initiate discussions with patients regarding CAM. Even when patients enquire about or request CAM for their AE, 34% pharmacists have said that they would only sometimes discuss this with the patient. A similar number (32%) would still never or rarely discuss this with patients even in the event of a patient requesting CAM. While 57% believed that patients should always be asked about their use of CAM, only 10% responded that they were always confident when talking to patients about CAM. Overall, half (50%) expressed some positivity with referring patients to CAM practitioners, whereas 43% were never or rarely happy to refer patients. These results are shown in Table 4.

**TABLE 4:** Durban community pharmacists’ practices regarding complementary and alternative medicines for atopic eczema.

Practices	Always (%)	Never (%)	Often (%)	Rarely (%)	Sometimes (%)
1. I initiate a discussion with patients regarding CAM for their AE.	7	31	9	23	29
2. I have a discussion when a patient requests CAM for their AE.	13	17	21	15	34
3. I ask about CAM use when taking a medication history for a new patient.	15	10	22	24	29
4. I am confident discussing CAM therapies with patients.	10	12	10	30	38
5. I believe that health practitioners treating patients for their AE should regularly ask patients if they are using CAM therapies.	57	1	29	2	10
6. I am happy to refer patients to CAM practitioners, for example, homeopaths, herbalists and so on for complementary treatment for their AE.	13	22	7	21	37

CAM, complementary and alternative medicines; AE, atopic eczema.

### Recommendations of complementary and alternative medicines

**FIGURE 1:**
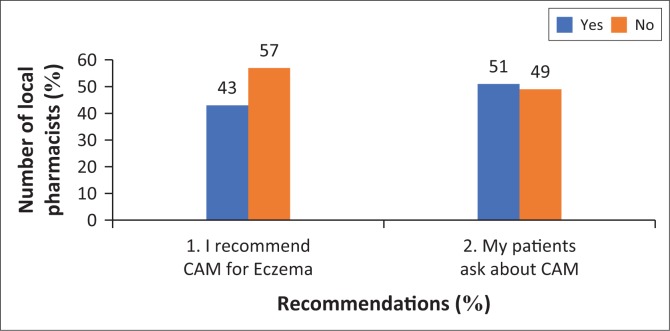
Percentage of local pharmacists that recommend complementary and alternative medicine (CAM).

Figure 1 shows that more than half of pharmacists (51%) are asked about CAM by their patients, and 43% (a substantial number) do recommend CAM for AE. These recommendations maybe based on enquiry from patients or pharmacists’ personal recommendations for AE.

### Knowledge and perspectives on education regarding complementary and alternative medicines

The perception of the current knowledge and education amongst pharmacists concerning CAM showed that 92% had never had or had minimal CAM training. Most (90%) do not access medical journals for information surrounding CAM, and 80% do not access information on the Internet. Only 9% of pharmacists frequently discussed the use of CAM for AE with other colleagues, while 43% reported rarely having these discussions. The majority (82%) reported that CAM was not discussed in any congress they have attended. Table 5 reflects these results.

**TABLE 5:** Durban community pharmacists’ education regarding complementary and alternative medicines.

Statement	No (%)	Rarely to minimal (%)	Frequent to substantial (%)
1. Did you have any training on CAM?	55	37	9
2. Do you access to any medical journals to source information on CAMs for AE?	44	50	6
3. Do you access any information on the Internet with regard to CAMs for AE?	35	45	20
4. Do you have discussions with other colleagues about CAM use for AE?	48	43	9
5. Is CAM for AE discussed in congresses you have attended?	82	15	4

CAM, complementary and alternative medicines; AE, atopic eczema.

**FIGURE 2:**
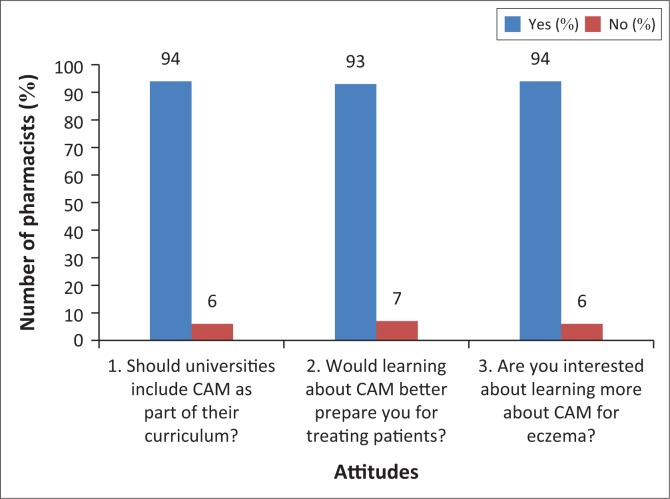
Durban community pharmacists’ attitude towards further education regarding complementary and alternative medicine.

The majority (94%) responded positively when asked if CAM education should be introduced into university curricula. Almost all (93%) agreed that CAM education would help them be better practitioners, and 94% showed a keen interest to further their knowledge on CAM. These results are shown in Figure 2.

## Discussion

The use of CAMs by patients suffering from AE has been documented globally (Ernst 2000) as well as in South Africa (Thandar et al. 2017). As such, most health care professionals are exposed to and interact with many patients either using CAMs or seeking advice on treatment. Pharmacists are the first line between patients and their medication and generally are the initial source of information regarding new and existing products. It would therefore be expected that they should be able to confidently advise patients regarding a large array of medications including CAMs. Because of the chronic nature of AE, and the large number of CAM products sold in pharmacies, as well as their extensive use amongst AE patients, this study assessed the knowledge, attitude and practices of pharmacists in a South African setting regarding the use of CAM for AE. The results have identified current information gaps that require urgent redress.

### Attitudes

The results showed a general positive receptiveness amongst pharmacists towards CAM. Other studies amongst pharmacists have also demonstrated a positive attitude (Easton 2007; Naidu et al. 2005). Despite having varied opinions, the majority in a study amongst rural community pharmacists in Australia agree that pharmacists have a role in ensuring that CAMs are used safely and effectively. Our study did not directly question the opinion of pharmacists regarding how they perceive their role in CAM; however, most agreed that health care practitioners need to be more informed on CAM, and there was also a notion that practitioners who were knowledgeable of CAM practices have better clinical outcomes. Another study amongst pharmacists in Australia described the use of CAM in conjunction with conventional medicine as ‘integrative care’ (Tiralongo et al. 2010). About half of community pharmacists in a study in Riyadh, Saudi Arabia, consider herbal remedies as unsafe, whereas the other half believed they were effective (Alkharfy 2010). In another South African study, more than 60% of pharmacists perceived herbs to be therapeutically effective drugs (Brijlal et al. 2011).

### Knowledge or familiarity

The pharmacists in our study showed little familiarity regarding the specific CAM practices. Probiotics and dietary supplements were the most well-known. Oral herbal products and topical herbal creams were less known but some familiarity was still reported. Homeopathy and CHM were the least known, most likely because the theory behind their methodology differs significantly from conventional medicine. Also, Durban itself has only a small Chinese population compared to other cities in South Africa, hence the limited exposure to Chinese practitioners and medicines. Another Durban-based study amongst health care workers in HIV and/or AIDS clinics reflected poor CAM knowledge including those of homeopathy and CHM (Mbutho, Gqaleni & Korporaal 2012). Australian pharmacists also reported a lack of knowledge about CAM and its safety and identified it as a definite barrier to their recommendations; however, they still did so together with conventional medicine as part of the pharmacy protocol (Culverhouse & Wohlmuth 2012).

### Communication

Pharmacists in our study indicated they do not generally initiate the conversation with patients over the use of CAMs. However, the pharmacists would discuss it when asked by the patients. This suggests that, currently, the patient is the initiator surrounding CAM discussions. This correlated well with another study conducted amongst pharmacists attending an International Pharmaceutical Federation (FIP) Congress and Traditional Chinese Medicine (TCM) Research Symposium, which included pharmacists working in retail and hospital pharmacies, that showed when CAM is discussed, the patient was more likely to have initiated the dialogue (Koh, Teo & Ng 2003). Koh et al. (2003:61) also noted that this lack of CAM dialogue represents a missed opportunity for the health care provider to provide a ‘teachable moment’ to patients. The results from our study showed that half of pharmacists were asked about CAMs by their patients; however, the problem that arises is that only 10% were always confident in discussing CAM therapies with patients. The absence of knowledge regarding their use plays a major role in this lack of confidence. Another South African study noted that the pharmacological knowledge of herbal medication was limited in pharmacists, and the respondents felt they were ill-equipped to advise patients to the best of their ability (Brijlal et al. 2011). In a local study (Mbutho et al. 2012), it was mentioned that a lack of communication between patient and health care practitioner on CAM choices could increase the potential risk of the patient because of exposure to adverse effects; and according to the WHO (2008), the lack of communication negatively impacts on the patient not making an informed decision regarding medical treatment options available to them (World Health Organization 2008). This is because they have partial, fragmented or unreliable information (Mbutho et al. 2012).

### Education

The lack of knowledge amongst pharmacists in our study is not only because of their lack of education or training regarding CAM in the undergraduate stage but also likely because of a limited desire for self-learning. It was clear in this study that very few sought CAM knowledge using accessible information sources. Most pharmacists rarely or never access medical journals or the Internet to source CAM information. Pharmacists have also said that CAM is not discussed in any congress they have attended, and very few even discuss CAM amongst their colleagues. This shows little interest in CAM amongst pharmacists despite many claiming to be keen to learn more. Considering that pharmacists are custodians of medicines and often the very first source of information for patients on CAMs, it is essential for pharmacists to be well informed on the uses, reported efficacies and safety of CAMs. Olatunde et al. (2010) supported this and added that pharmacists should have at least a basic knowledge of CAM much like all other over-the-counter medication to properly counsel patients. It was further reported that all respondents also felt pharmacists play a key role in safety monitoring with regard to contraindications (Olatunde et al. 2010). A study in Australia added that most perceived barriers are owing to a lack of knowledge and the prime concern of pharmacists was the safety of patients. When the level of comprehensiveness increased, pharmacists were less likely to sell products that were inappropriate for the patient (Kanjanarach et al. 2011). This lack of knowledge of pharmacists points to gaps in current pharmacy curricula and the need to reassess the education to improve clinical outcomes, especially in chronic diseases like AE on which patients consistently require counsel.

Our results showed that the majority of pharmacists agree with the sentiment of CAM being formally introduced into the university curricula. In an Australian study, similar feedback was received by pharmacists who stated that one of the reasons they do not recommend CAM is because they felt inadequately trained (Culverhouse & Wohlmuth 2012). In a study of pharmacy graduates from the University of KwaZulu-Natal, Govender et al. (2009) showed that only 26.5% of respondents felt adequately competent with their knowledge of CAM (Govender et al. 2009). The study further reported that of the pharmacy graduates who felt comfortable discussing traditional healers, in the South African context, only 4.6% indicated the training was obtained from the university (Govender et al. 2009). The majority of respondents of our study agreed that better CAM education would make them better practitioners and wanted to further their education in CAM.

## Limitations of the study

Although we have compared the knowledge, attitude and practices of the pharmacists in this study to those in other studies, this study specifically looked at CAM for AE, whereas other studies were on the general use of CAM. We have presumed that the pharmacists’ responses regarding AE would be similar to those on general CAM use. The responses by pharmacists in this study can not be generalised and may not represent the views of all South African pharmacists, considering our study sample, which includes a majority of Indian pharmacists within the Durban metropolitan area.

## Conclusion

It is evident that CAMs are used by a large part of the population. When they are used together with conventional medicine (Magin & Adams 2007; Noiesen et al. 2007), it may be difficult to ascertain therapeutic benefits from either type of medicine and identify adverse effects and drug interactions. The combination of CAM with conventional medicines can affect the overall clinical outcome as this often leads to non-compliance with prescribed therapy. Pharmacists therefore need to realise and accept responsibility in understanding whether there is any evidence-based role for the use of CAM. They should also become more conversant with common CAM therapies and ensure that their use is safe. This will strengthen communication with patients and help influence better patient outcomes. Our study uncovered poor CAM knowledge and communication between pharmacist and patient, but a strong interest amongst them to learn more. The researchers further identified the need for continuing education programmes in CAM for practising pharmacists, as well as introducing CAM training in the undergraduate curriculum.
